# Impact of 4-epi-oxytetracycline on the gut microbiota and blood metabolomics of Wistar rats

**DOI:** 10.1038/srep23141

**Published:** 2016-03-15

**Authors:** Hongxing Han, Hailong Xiao, Kai Zhang, Zhenmei Lu

**Affiliations:** 1 College of Life Sciences, Zhejiang University, 866 Yuhangtang Road, Hangzhou 310058, China; 2 Hangzhou Institute for Food and Drug Control, Hangzhou 310004, China

## Abstract

The impact of 4-epi-oxytetracycline (4-EOTC), one of the main oxytetracycline (OTC) metabolites, on the gut microbiota and physiological metabolism of Wistar rats was analyzed to explore the dynamic alterations apparent after repeated oral exposure (0.5, 5.0 or 50.0 mg/kg bw) for 15 days as shown by 16S rRNA pyrosequencing and UPLC-Q-TOF/MS analysis. Both principal component analysis and cluster analysis showed consistently altered patterns with distinct differences in the treated groups versus the control groups. 4-EOTC treatment at 5.0 or 50.0 mg/kg increased the relative abundance of the Actinobacteria, specifically Bifidobacteriaceae, and improved the synthesis of lysophosphatidylcholine (LysoPC), as shown by the lipid biomarkers LysoPC(16:0), LysoPC(18:3), LysoPC(20:3), and LysoPC(20:4). The metabolomic analysis of urine samples also identified four other decreased metabolites: diacylglycerol, sphingomyelin, triacylglycerol, and phosphatidylglycerol. Notably, the significant changes observed in these biomarkers demonstrated the ongoing disorder induced by 4-EOTC. Blood and urine analysis revealed that residual 4-EOTC accumulated in the rats, even two weeks after oral 4-EOTC administration, ceased. Thus, through thorough analysis, it can be concluded that the alteration of the gut microbiota and disorders in blood metabolomics are correlated with 4-EOTC treatment.

The human gut microbiota, which is ten-fold greater in cell number than all of our somatic cells, has an inconceivably complex structure and a bewildering variety of compositions^1^,^2^. This community has coevolved with living organisms, manipulating and complementing the biology of the human body in mutually beneficial ways^3^. The human “metagenome” consists of *Homo sapiens* genes and genes present in the colonizing microbes, which endow people with functional features that we have yet to refine as a species^4^. The gut microbiota has been recognized as a significant factor in determining host health and conferring extended metabolic capacities on the host^5^,^6^ due to its influence on whole-body metabolism, its alteration of the energy balance, and the metabolic inflammation associated with chronic diseases and related disorders^7^,^8^. Furthermore, recent studies have demonstrated that the structure of the gut microbiota, specifically the relative abundance of dominant bacterial divisions, such as the Firmicutes and Bacteroidetes, play vital roles in obesity and metabolic diseases such as type-2 diabetes, angiocardiopathy and nonalcoholic fatty-liver disease^9^,^10^,^11^. The gut microbiota may be commonly affected by a variety of external factors, specifically food intake and medicinal disturbance, which may further affect host health^1^,^12^.

Of most concern among these multiple factors are antibiotics; an instrumental tool in controlling pathogen infections, these metabolites are designed by nature to target and inhibit microorganisms in a variety of ways. The majority of clinical antibiotics have broad spectrums of activity; hence, in addition to killing pathogenic bacteria, they can also potentially inflict collateral damage on commensal bacteria in the gut, including those with health-promoting roles such as *Bifidobacterium* and *Lactobacillus*^13^. Rifaximin, a non-absorbable antibiotic, can strikingly increase the abundance of lactobacilli in the ileum^14^,^15^. Moreover, the spread of antibiotic resistance in pathogens, which is facilitated by transposons and common between mutualists^16^, has generated enormous concern related to the negative impact of antibiotics, specifically with respect to the increasing frequency of antibiotic resistance genes (ARGs) in the gut microbiota^17^. Conclusive evidence has revealed that energy metabolism, particularly the tricarboxylic acid cycle and indoxyl-sulfate excretion, is altered after penicillin and streptomycin sulfate treatment^6^. Additionally, bile acid metabolism in adult mice, as well as their intestinal bacteria, was distinctly altered after treatment with various antibiotics including vancomycin, ciprofloxacin, neomycin, and aztreonam^18^. As such, the sophisticated relationship between antibiotics and the bacterial community in the gut has not been clearly elucidated.

Although a certain portion of absorbed antibiotics can be eliminated through bodily excretion, the residues and their metabolites have been shown to remain in organisms at high concentrations over an extended period of time. Oxytetracycline (OTC) is one of the most commonly used tetracyclines for controlling and preventing bacterial infections^19^, and its main degradation product, 4-epi-oxytetracycline (4-EOTC), is frequently detected in agricultural products and ecological settings^20^,^21^,^22^,^23^,^24^. It has been reported that OTC treatment reduces the bacterial diversity of the intestinal microbiota and facilitates the proliferation of opportunistic pathogens in Atlantic salmon^25^. Although 4-EOTC was reported to possess a similar, if not higher, toxicity as that of its parent chemical^26^, few studies have focused on the safety of OTC metabolites. 4-EOTC may pose a serious threat to human health and to the environment, and it is necessary to evaluate the safety of 4-EOTC for any potential risks pertaining to public health by comprehensively understanding OTC application.

Through previous studies, we have found that 4-EOTC can induce toxicity and metabolomic changes in Wistar rats after short-term, repeated oral administration^27^. The purpose of this study was to systematically explore the metabolomic impacts of 4-EOTC on the gut microbiota and host metabolism. We hypothesized that not only the antibiotics but also their major metabolites may pose a serious threat to human health and the environment because antibiotic metabolites generally possess the same or even higher toxicities as those of their parent chemicals. Thus, such metabolites may affect public health, especially through the gut microbiota and/or blood metabolites. We addressed these hypotheses through the analysis of the rat gut microbiota via 16S rRNA gene sequencing and metabolite profiling. These results are of great importance for human health and should draw attention to the impacts and potential risks of antibiotic metabolites in food and the environment.

## Results

To assess the impact of 4-EOTC on the gut microbiota and blood metabolomics of Wistar rats, we collected fecal and blood samples during and after a 15-day 4-EOTC intervention at different dose levels. The extracted DNA was sequenced using next-generation sequencing technology, and the relative abundance of ARGs was estimated through quantitative PCR (Q-PCR). We also used UPLC-Q-TOF/MS analysis to evaluate the metabolic profiles of the different treatment groups.

### Overall structural changes and taxonomic analysis of structural shifts in the gut microbiota in response to 4-EOTC treatment

A total of 4,686,642 usable pyrosequencing reads (10,016 unique sequences) were obtained from 64 samples. After discarding sequences that had no near-neighbors in the entire RDP database, 3,486,574 reads (an average of 54,477 sequences per sample) were delineated using mothur (version 1.31.2, http://www.mothur.org/) into 2,683 operational taxonomic units (OTUs) at the 97% similarity level. Rarefaction and Shannon diversity curves revealed that although new rare phylotypes would be expected with additional sequencing, most of the diversity had already been captured (Figure S1).

**Figure 1:**
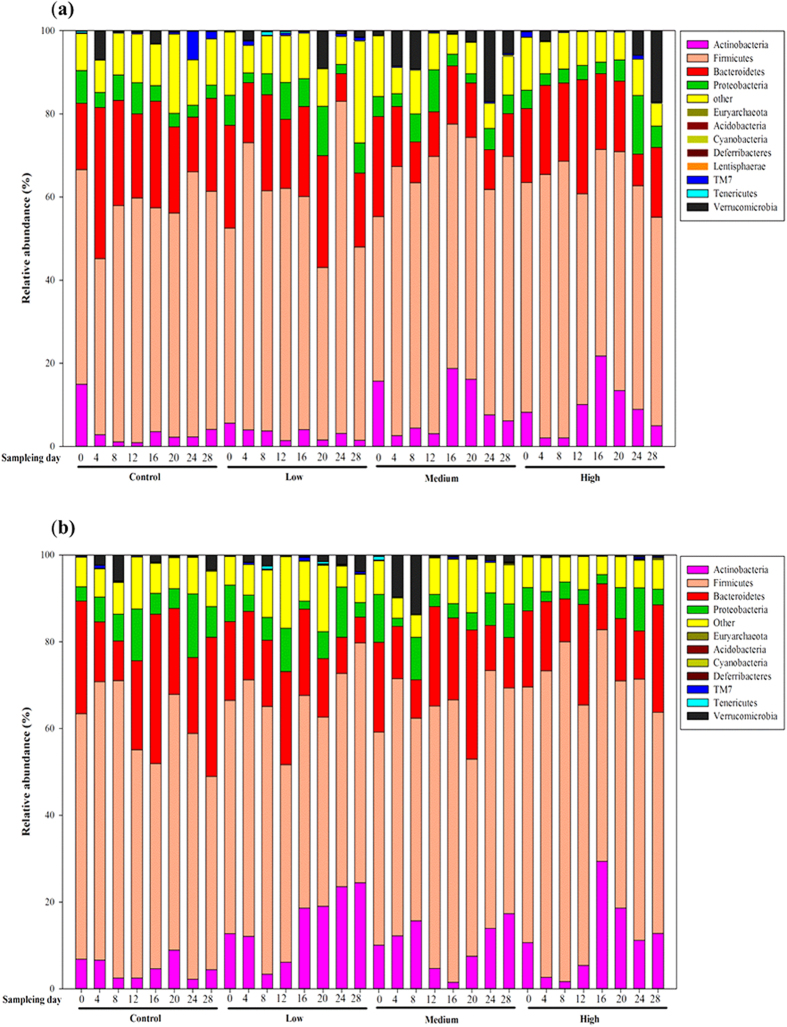
The relative abundances of the gut microbiotas (at the phylum level) in different groups at different time points in males (**a**) and females (**b**).

The different phyla were assessed by taxonomic assignment of all the sequences using RDP Classifier. Among all the interpretable sequences, Firmicutes was the predominant phylum, varying from 41.5% to 79.9% of the gut microbiota in both the treated and control groups, followed by Bacteroidetes, which contributed from 7.6% to 36.4% in the different groups. After 15 days of 4-EOTC treatment, the relative Firmicutes content only increased slightly in both genders. The level of the Bacteroidetes was also altered after the 15-day treatment, from 25.63% in the control males to 18.21% in the high-dose males. The alteration was more drastic in females, decreasing from 34.42% to 10.59%. The relative levels of the Actinobacteria drastically increased on day 16 after 4-EOTC treatment, which was 3.56% in the control males versus 18.77% and 28.77% in the medium- and high-dose males (Fig. 1). In the low-dose female groups, by day 28, the abundance of the Actinobacteria increased to as much as 24.43% in the gut microbiota, whereas their abundance was only 4.41% in the control group. These phenomena were sustained for at least two weeks in our study. The increased abundance of Verrucomicrobia in the medium- and high-dose male groups, which was 17.03% and 17.11%, respectively, indicated a structural disturbance due to 4-EOTC 12 days after treatment. Thus, we deduced that 4-EOTC changed the structure of the gut microbiota, specifically the relative abundances of the Actinobacteria, Bacteroidetes and Firmicutes phyla.

**Figure 2:**
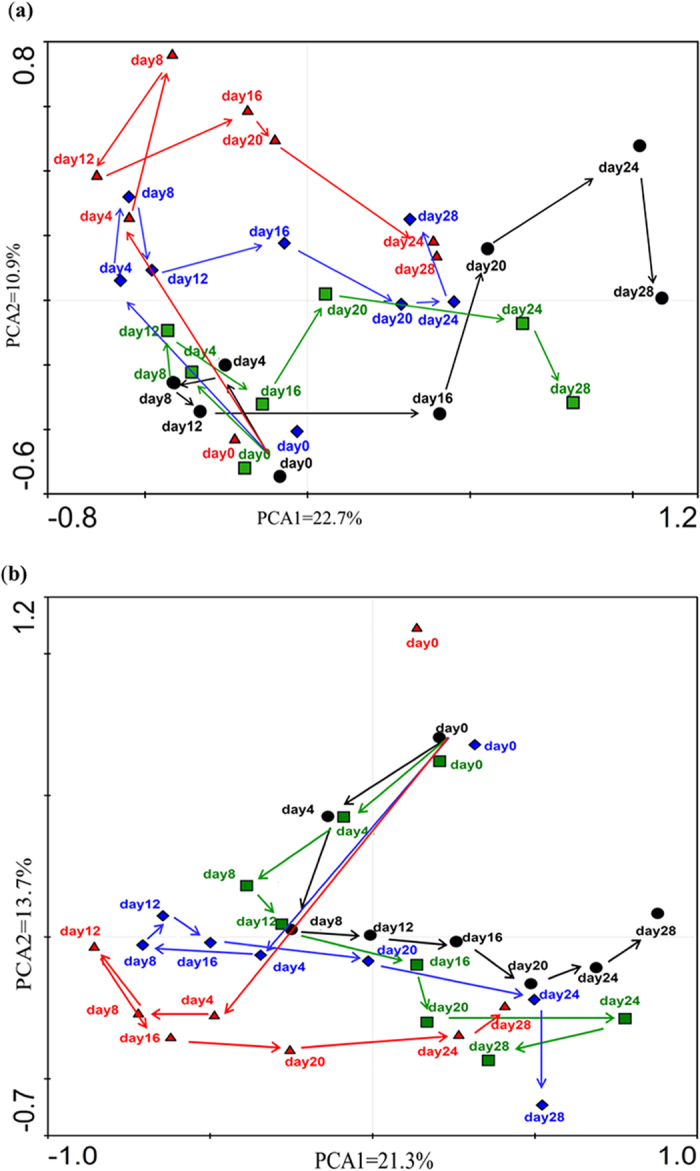
PCA analysis of the gut microbiotas of different groups of males (**a**) and females (**b**) at different time points. Arrows represent the trajectory change of gut microbiota structures in different groups. •: control groups; 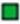: low-dose groups; 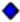: medium-dose groups; 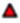: high-dose groups.

A Principal Component Analysis (PCA) based on the relative abundance of OTUs and evolutionary relatedness revealed distinct gut microbial communities in the control versus treated groups, as revealed by two PC scores calculated using the Canoco software for Windows (version 4.5). The two PCs accounted for 33.6% and 34% of the total variance exhibited by the different OTUs, whereas dose levels in males and females revealed the same patterns of fluctuation in both genders. During the 4-EOTC treatment period, the points of the medium- and high-dose groups were distinct from those of the low-dose and control groups (Fig. 2). The trajectory of the groups after 4-EOTC treatment ceased suggested that the gut microbiota was resilient, as the composition of the gut microbiotas of the medium- and high-dose groups approached that of the control. However, this structural recovery was limited, as evidenced by the continued abnormal state of the treated groups after 2 weeks.

**Figure 3:**
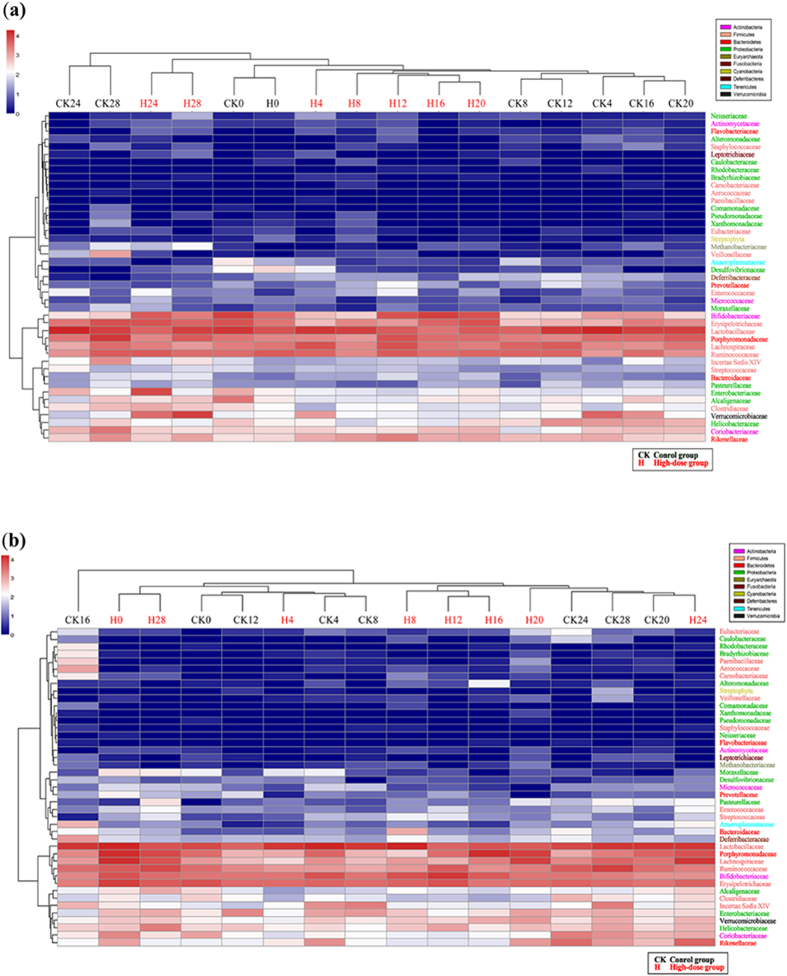
Cluster analysis and heat-map of high-dose males (**a**) and females (**b**) compared with control groups as constructed with the R statistical software (2.15.2). CK: control group; H: high-dose group. The number following “CK” or “H” indicates the sampling time.

Cluster analyses were performed to compare the overall structures of the gut microbiotas of all the samples and to determine whether any specific bacterial phylotypes were associated with 4-EOTC treatment. Forty-three identifiable families were selected as key variables filtered from the confirmed OTUs (Table S2), and the cluster analysis of all the groups showed apparent differences between the high-dose groups and the control, whereas no obvious distinction was observed between the medium- or low-dose groups and the control (Figure S2). We further compared the high-dose groups and the control individually (Fig. 3): 14, 5, 14 and 4 of the OTUs were assigned to the phyla Proteobacteria, Bacteroidetes, Firmicutes and Actinobacteria, respectively. The other eight families identified belonged to six other phyla. The cluster of the treated groups during periods of treatment decreased compared with that of the control in both males and females, demonstrating that 50 mg/kg bw 4-EOTC distinctly altered the normal structural composition of the gut microbiota. Compared with those of the control group, the relative levels of Bifidobacteriaceae, Enterococcaceae, and Actinomycetaceae increased significantly, whereas the levels of Lactobacillaceae, Aerococcaceae, Helicobacteraceae, and Pasteurellaceae dramatically decreased, in high-dose males after treatment with 4-EOTC for 15 days. In females, 4-EOTC also increased the relative levels of Deferribacteraceae and Lachnospiraceae, but not that of Bifidobacteriaceae. In addition, the relative abundance of Lactobacillaceae also decreased from 39.38% to 16.17%, more than half that of the control, which was similar to the levels in the males. Eubacteriaceae, Micrococcaceae, and Helicobacteraceae also decreased as a result of 4-EOTC treatment (Table S2).

### Quantitative PCR of ARGs

**Figure 4:**
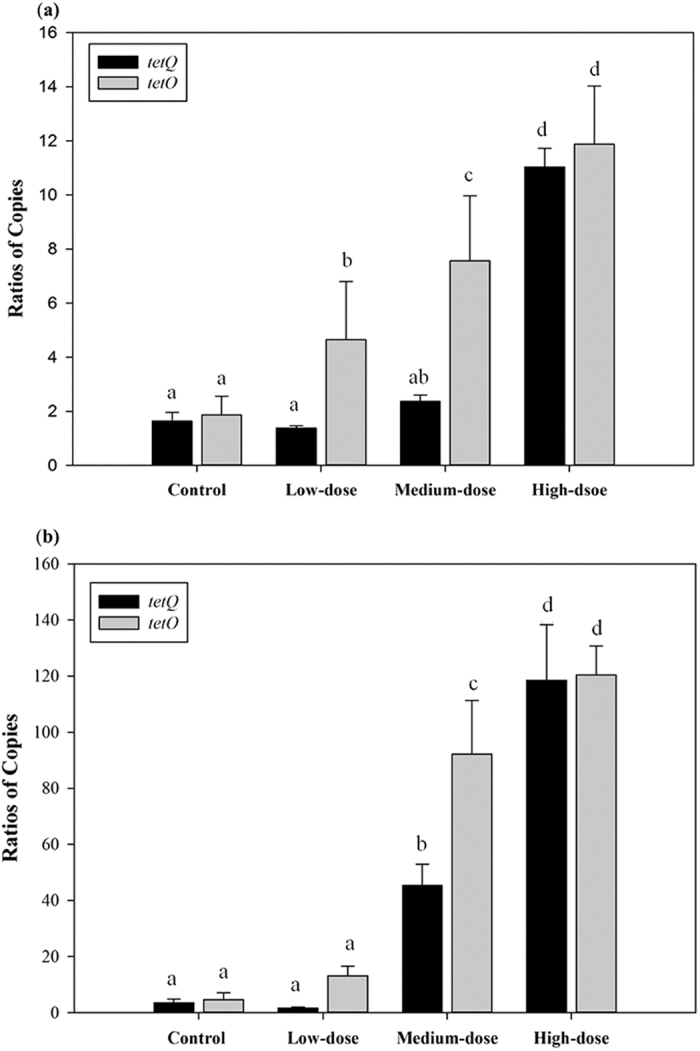
Relative abundances of the genes *tetQ* and *tetO* obtained from qPCR in the males (**a**) and females (**b**) of the four groups at day 16. All data are presented as the mean ± SE. Different letters designate significant difference (*p* < 0.05).

Q-PCR was used to determine the relative amounts of antibiotic resistance genes in all samples after 4-EOTC treatment (Fig. 4). The results showed that 15 days of 4-EOTC administration caused a detectable increase in the abundance of ARGs. *TetO* was found to be significantly more abundant in high-dose rats than in control rats (*p* < 0.01), as well as *tetQ* (*p* < 0.01, male; *p* < 0.05, female). This phenomenon was also observed in the medium-dose 4-EOTC-treated females (*p* < 0.05). The relative ratios of ARGs indicated that the intake of certain amounts of 4-EOTC raised the percentage of resistant strains by increasing the frequency of ARGs.

### Trajectory analysis of residual 4-EOTC in blood and urine samples

**Figure 5:**
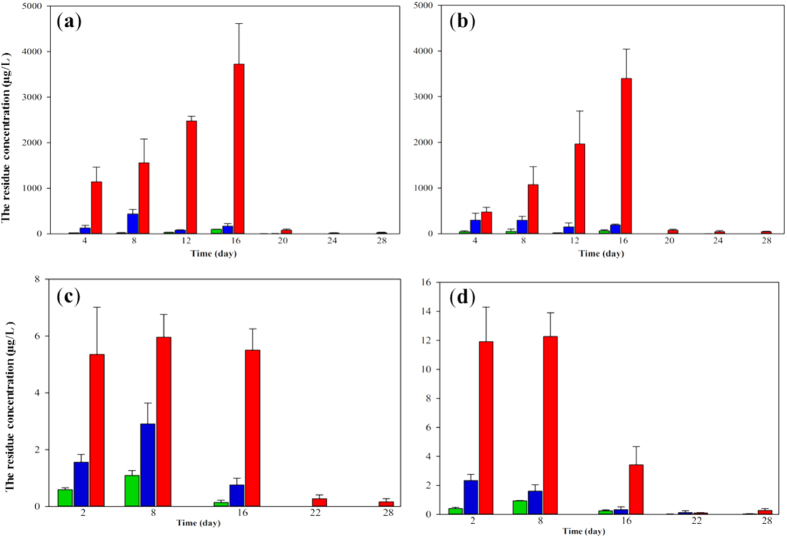
The residual concentration of 4-EOTC in urine and blood samples during the study period, as assessed by HPLC-MS. (**a**) male urine; (**b**) female urine; (**c**) male blood; (**d**) female blood. Green column: low-dose group; blue column: medium-dose group; red column: high-dose group. All data are presented as the mean ± SE.

Chromatographic analysis highlighted the specificity of the assays, resulting in no observed interferences in the regions of elution. The calibration curves for the urine and blood samples were y = 0.8995x + 0.0341 (R^2^ = 0.9994) and y = 0.8986x + 0.1317 (R^2^ = 0.9991), respectively, with detection limits of 4.5 μg L^−1^ and 0.9 μg L^−1^, respectively. The residual 4-EOTC present during the administration period and the withdrawal period in the urine and blood samples is shown in Fig. 5. 4-EOTC could be detected in urine samples 4 days after treatment and accumulated over the course of administration. The concentration in the urine samples of high-dose male rats reached approximately 4,000 μg L^−1^, indicating that most of the chemical was eliminated through the urine. However, residual 4-EOTC could still be detected at approximately 30 μg L^−1^ in urine samples after treatment ceased 14 days later. With respect to the blood samples, residual 4-EOTC in high-dose males and females was approximately 5 μg L^−1^ and 3 μg L^−1^, respectively. However, during administration, residual 4-EOTC reached as high as 19.06 μg L^−1^ in female blood samples. Moreover, 4-EOTC was still detected in blood samples 15 days after administration was stopped in the medium- and high-dose groups. This results suggested that a certain amount of 4-EOTC remained in the blood or tissues after absorption by the body. Although the residual 4-EOTC may be metabolized some time later, it may still affect physiological metabolism in certain ways.

### UPLC-Q-TOF/MS metabolomic analysis of blood samples after 15 days of 4-EOTC treatment

**Figure 6:**
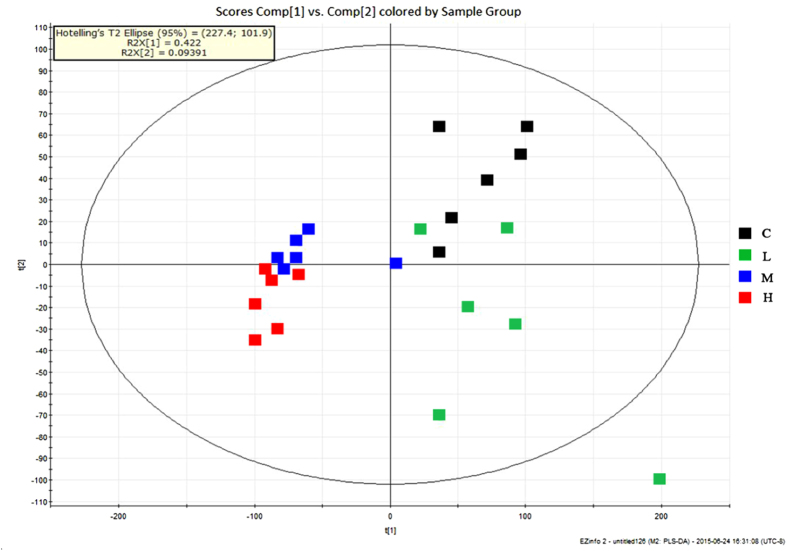
PLS-DA score plot of mice blood samples after a 15-day 4-EOTC treatment based on UPLC-TOF/MS in the positive mode (n = 6). C: control group; L: low-dose group; M: medium-dose group; H: high-dose group.

The blood samples on day 16 from half of each male and female groups were investigated using UPLC-Q-TOF/MS in the positive-ion mode. The supervised partial least squares discriminant analysis (PLS-DA) model was built using data from all groups to determine the metabolic differences and metabolite profiles of the control and treated groups. As shown in Fig. 6, the low-, medium-, and high-dose groups were distinct from the control groups after the 15-day 4-EOTC treatment. Potential biomarkers played important roles in this separation and were evaluated using the Variable Importance in the Project (VIP) parameter (VIP > 1), which is a major parameter in defining the PLS-DA model. Table 1 shows the corresponding retention times, m/z values, proposed elemental compositions and VIP values of the 14 candidate metabolites preliminarily identified from the chosen ion mode. These biomarkers were identified according to the accurate masses measured by UPLC-Q-TOF/MS compared to the theoretical masses, as well as the fragment ions in the Human Metabolome Database (HMDB; METLIN). A total of 4, 12 and 14 metabolites were significantly changed in the low-, medium- and high-dose treated groups, respectively (*p* < 0.05). The observed elevation of the LysoPCs and DG(34:5) induced by 4-EOTC was significant in the medium- and high-dose groups, which indicated potential hepatic damage and enzyme disorders in the treated rats. Simultaneously, 4-EOTC also decreased the content of SMs, PGs and TG in the two groups. The concentrations of 5-L-glutamylglycine, arginyl-serine and deoxyhypusine were all diminished in the treated groups. The alteration of these biomarkers demonstrates the potential biological effects induced by 4-EOTC treatment in animals.

## Discussion

Although antibiotics have irreversibly altered the field of modern medicine and significantly improved the quality of life for countless individuals, we are apprehensive that these life-altering drugs did not arrive without imposing some collateral damage on the gut microbiota. These bacteria interact extensively with the host through the metabolic exchange and co-metabolism of substrates to maintain the normal function and health of the intestinal tract and, ultimately, the entire body. In this study, we revealed that 4-EOTC, the main product of OTC metabolism, can accumulate in organisms and alter the structural composition of the gut microbiota, thus affecting physiological metabolism. In our previous study, we observed the toxic effects and urine metabolomic changes induced by this chemical^27^. This study focused on the trajectory of residual 4-EOTC present in blood and urine samples and its detailed effects on the gut microbiota.

Recent metagenomic and pyrosequencing studies on intestinal microbiota have highlighted the yet-undiscovered diversity of phylotypes present and reshaped the expected proportional abundance of known phyla, particularly higher levels of Actinobacteria than previously estimated^28^,^29^. Increased levels of Actinobacteria are associated with the fecal microbiotas of obese subjects^30^, indicating the importance of this phylum. In this study, 4-EOTC treatment was the main factor that affected the composition and structure of the gut communities. The abundance of Actinobacteria, specifically Bifidobacteriaceae, increased in both genders, revealing the structural changes induced by 4-EOTC treatment. The Bifidobacteriaceae, which were further determined to consist of *Bifidobacterium*, play a vital role in carbohydrate metabolism^31^. Bifidobacteriaceae provide well-known benefits to human health and have been proven to decrease intestinal endotoxin levels and improve mucosal barrier function^32^,^33^. The relative contents of Lactobacillaceae and Helicobacteraceae were both significantly decreased in the treated groups. Members of the family Lactobacillaceae, which are responsible for the secretion of lactic acid as the major end product in carbohydrate metabolism, have vitally important roles in normal gut bacterial communities. This study revealed that the type of carbohydrate metabolism performed by the gut microbiota was altered after 4-EOTC treatment.

In addition, the Helicobacteraceae are frequently associated with certain diseases such as diarrhea, inflammatory bowel diseases, hepatitis and hepatic cancer^34^,^35^. It is commonly acknowledged that OTC antibiotics can be used to promote growth, as well as kill certain bacteria^36^. From the changes induced by 4-EOTC treatment, it is apparent that the OTC metabolite 4-EOTC may increase the relative abundance of the Bifidobacteriaceae and decrease that of the Lactobacillaceae in the gut microbiota as a means of improving energy intake and thus promoting growth. By changing the composition and functionality of the microbiota, 4-EOTC can facilitate the competitive exclusion of potential pathogens such as the Helicobacteraceae and Enterococcaceae. Further analysis is required to provide detailed molecular links among the patterns detected in this work.

Both a PCA analysis and a cluster analysis with a heat-map demonstrated that high-dose 4-EOTC treatment altered the structural composition of the gut microbiota in the current study. Although the gut ecosystem has certain restorative forces, the microbial community was not completely restored in all cases after 4-EOTC treatment (even two weeks after treatment ceased). Even in the low-dose treatment group, a slight disturbance exerted persistent effects on the structural composition of the gut microbiota for at least 15 days. After perturbation by antibiotics, the structure of the gut microbiota began to recover to its initial state 1 week after the end of each antibiotic treatment; however, full recovery was not apparent until approximately 5 months later^17^. Our results reveal that 4-EOTC treatment may induce a structural shift in the gut to an alternative stable state that is unlikely to revert quickly to the original state, resulting in near-irreversible bacterial community shifts (such as in the Bifidobacteriaceae and Lactobacillaceae). These communities play instrumental roles in keeping individuals healthy. Though the PCA score plots of the medium- and high-dose groups showed tendencies similar to that of the control groups, the cluster analysis revealed the differences among these groups.

In gut communities, the survival and influence of pathogens and opportunistic pathogens depends on antibiotic resistance^37^. Antibiotic resistance can be achieved by the acquisition of mobile genetic elements through horizontal gene transfer, and antibiotic resistance genes are closely associated with transposons, plasmids, and integrons^38^,^39^. For example, the *tet* genes coding for tetracycline efflux proteins are normally part of plasmids in water environments^36^,^40^. Monitoring the number of copies of *tetO* and *tetQ* revealed that 4-EOTC treatment increased the abundance of these antibiotic resistance genes, which could then be transferred and spread in the gut microbial communities. Residual 4-EOTC also accumulated in the blood and tissues for a certain time period, as indicated by the trajectory analysis of 4-EOTC. This accumulation poses a potential threat to public health and deserves further attention.

In this study, 14 potential biomarkers were identified from the UPLC-Q-TOF/MS analysis. The biological functions and metabolic pathways associated with these metabolites were investigated using databases such as HMDB and KEGG, which are available electronically and can be used for querying metabolic pathways and contain some metabolomic information. Lysophosphatidylcholines (LysoPCs), which are generated by phospholipase A_2_, have been reported to play important roles in angiocardiopathy and other hepatic diseases^41^,^42^,^43^. By using a dynamic monitoring strategy, we showed that the concentrations of LysoPC(18:3), LysoPC(20:3) and LysoPC(20:4) all increased significantly after 4-EOTC treatment. PG(16:0/16:1(9Z)), PG(16:1/22:6), SM(d18:0/14:0), SM(d18:0/22:3(10Z, 13Z, 16Z)), TG(14:0/15:0/16:0), and the LysoPCs all have essential functions in the lipid metabolism of organisms^44^,^45^,^46^. It can be concluded that normal lipid metabolism was apparently changed after 4-EOTC treatment, particularly in the medium- and high-dose groups.

5-L-Glutamylglycine and arginyl-serine are dipeptides that are commonly produced from polypeptides by the action of the enzyme dipeptidyl peptidase in protein catabolism. Deoxyhypusine is a substrate of deoxyhypusine synthase, which catalyzes the cleavage of the polyamine spermidine and the transfer of its 4-aminobutyl moiety to the ε-amino group of one specific lysine residue of the eIF-5A precursor^47^. 4-EOTC reduced the concentration of these three compounds significantly, indicating that amino acid metabolism was altered in all treated rats. In addition, decreases in 4-methylthio-2-oxobutanoic acid, a potent inducer of apoptosis in a BAF3 murine lymphoid cell line^48^, may indicate inflammation in the medium- and high-dose rats. The previous observed histopathology results also support such pathological effects.

In conclusion, 4-EOTC is taken up through the intestinal tract, absorbed into the blood, and excreted through urine. This antibiotic metabolite altered the composition and function of the gut microbiota, and the disturbed structure of the gut microbiota did not completely recover after removing this stress, indicating the damaging effects of this compound. The relative levels of antibiotic resistance genes also increased in gut communities after treatment. Furthermore, 4-EOTC was present in blood and tissue samples even two weeks after chemical administration had ceased. The blood metabolomic changes showed that metabolism was altered drastically in the 4-EOTC-treated groups. These results provide tangible proof that antibiotic metabolites may also affect human health through complex pathways. Potential changes to the gut microbiota and metabolomics should be seriously considered during safety assessments, and the safety of antibiotic metabolites should also be comprehensively reevaluated.

## Methods

### Study design

Healthy male and female Wistar rats, aged 5 weeks and weighing 140–150 g, were purchased from the Shanghai Animal Center. Standard rat food and sterile water were available *ad libitum*. The animals were housed in plastic cages with stainless steel wire covers under standard conditions at a temperature of 22 ± 2 °C, a relative humidity of 55 ± 10%, and a light/dark period of 12/12 h. All animals were acclimated for 5 d before the treatment. The protocol was approved by the Council of Animal Care of Zhejiang University (No. ZJU 201406-1-02-055), and all procedures in this study were performed according to their guidelines.

The 4-EOTC was prepared in normal saline (9 g NaCl dissolved in 1,000 mL Milli-Q water) 2 h before each administration. The rats were randomly divided into the following four groups (12 rats each, 6 males and 6 females): control, low-dose, medium-dose and high-dose, the latter three of which were administered with 4-EOTC in normal saline at 0.5 mg/kg bw, 5.0 mg/kg bw and 50.0 mg/kg bw, respectively, for 15 consecutive days. Fecal and urine samples were collected from each of the four groups every 4 days on days 0 (the day before administration), 4, 8, 12, 16, 20, 24, and 28. All fecal samples were stored at −80 °C until analysis. The urine samples were centrifuged at 8,000 × g for 10 min at 4 °C, and the obtained supernatants were stored at −80 °C. Whole blood was sampled from all rats on days 2, 8, 16, 22, and 28. The blood samples were immediately mixed with 10% TCA containing 1 mM EDTA in a 1:1 (v/v) ratio and stored at −80 °C until analysis.

### Fecal DNA extraction and pyrosequencing

Metagenomic DNA was extracted from the feces of each group using the MO BIO PowerFecal™ DNA Isolation Kit (MOBIO, USA) according to the manufacturer’s instructions. The DNA concentration was measured using a Nanodrop-2000 (Thermo Scientific). PCR amplification of the V4 region of the 16S rRNA gene was performed using the forward primer 5′-AYTGGGYDTAAAGNG-3′ and the reverse primer 5′-TACNVGGGTATCTAATCC-3′^49^. The PCRs were completed under the following conditions: a pre-denaturation at 98 °C for 4 min; 27 cycles of 98 °C for 30 s, 50 °C for 30 s, and 72 °C for 30 s; a final extension at 72 °C for 5 min; and a hold at 4 °C. The PCR mixtures had a final volume of 25 μL consisting of 5 μL Q5 Reaction Buffer, 5 μL Q5 high-GC Enhancer, 2 μL dNTPs, 1 μL forward primer (10 μM), 1 μL reverse primer (10 μM), 1 μL template DNA, and sterile PCR-grade water. All samples were assayed in duplicate. The PCR products were analyzed by 0.8% agarose gel electrophoresis. Only PCR products without primer dimers or contaminating bands were used for sequencing by synthesis. Sequencing was performed by the Personal Biotechnology Co., Ltd. (Shanghai, China) via an Illumina MiSeq instrument.

### Bioinformatics and multivariate analysis of pyrosequencing

In addition to standard sequence quality control, raw sequencing reads filtered by the instrument software were removed if they 1) did not include a correct primer sequence, 2) were shorter than 150 bp (excluding the primer sequence) or 3) included any ambiguous bases. Only sequences with an overlap longer than 10 bp and without any mismatch were assembled. High-quality sequences were uploaded to QIIME for further study^50^. The 150-bp trimmed pyrosequencing reads were aligned and clustered into OTUs with complete linkage clustering at a maximum within-cluster distance of 3% using the RDP pyrosequencing pipeline (Release 11.1, http://rdp.cme.msu.edu/)^51^,^52^. Rarefraction analysis on the fecal microbiota was performed using Analytical Rarefraction 1.3 (http://www.uga.edu/strata/software/). The abundance and diversity indices were generated using mothur with an OTU identity cutoff of 97% after implementing a pseudo-single linkage algorithm^53^.

### Real-time quantitative PCR of ARGs

To verify the abundances of ARGs after 4-EOTC treatment, two typical ARGs (*tetQ* and *tetO*) were selected and measured by Q-PCR^36^,^54^. The plasmids containing the target genes were obtained by molecular cloning. Primers and related information for the ARGs and 16S rRNA are shown in Table S1. The PCR protocol was denaturation at 94 °C for 5 min, followed by 35 cycles of denaturation at 94 °C for 30 s, annealing at a given temperature (Table S1) for 45 s and extension at 72 °C for 45 s, followed by a final extension at 72 °C for 10 min. The PCR products were analyzed by gel electrophoresis and further confirmed by DNA sequencing. Duplicate reactions were performed for each permutation of samples and primer sets to ensure reproducibility. Additionally, sterile water was used as a negative control.

The Q-PCR was performed in a Rotor-Gene Q Machine with the Rotor-Gene Q Software 2.1 (QIAGEN, Germany) using the following protocol: 95 °C for 5 min, followed by 40 cycles of denaturation at 95 °C for 10 s and annealing at 60 °C for 30 s. Each reaction was conducted in quadruplicate. Calibration curves were generated using a 10-fold serial dilution of the plasmid containing the target genes. Eubacterial 16S rRNA genes were quantified simultaneously to minimize the variation of extraction efficiency, and the relative abundances of the target genes were normalized to each total bacterial community.

### Sample preparation and HPLC-MS

Prior to analysis, blood samples were thawed, diluted 1:2 with acetonitrile, and centrifuged at 4,000 × g for 10 min at 4 °C. The supernatant was extracted with SPE columns and syringe filter units (0.22 μm) for HPLC–MS and UPLC-Q-TOF/MS analyses^55^,^56^,^57^. Thawed urine samples were directly extracted using SPE columns and syringe filter units (0.22 μm) before injection^58^.

Residual 4-EOTC in all blood and urine samples was analyzed by HPLC-MS. Liquid chromatography was performed using an Agilent 6410A HPLC system (Agilent Technologies, Palo Alto, CA, USA) equipped with a cooled autosampler-controlled gradient system. The analytical column used was an XDB-C18 (Agilent Technologies, 927975-902) chromatographic column (50 × 4.6 mm ID, particle size 1.8 μm). The separation was performed by gradient elution. Mobile phase A contained 0.1% formic acid water, and mobile phase B was acetonitrile. The gradient elution was conducted as follows: 0–1 min, 90% A and 10% B; 1–3 min, a linear gradient to 90% A and 10% B; 3–3.2 min, 40% A and 60% B; 3.2–6 min, a linear gradient to 90% A and 10% B. The injection volume was 2 μl, and the flow rate was 0.4 mL min^−1^. The HPLC system was connected to a Sciex API 3000™ (Applied Biosystems, Foster City, CA, USA) equipped with an electrospray source. The instrument was operated in the positive mode, and the temperature of the heated capillary was 350 °C. The source voltage gas was set at 4.2 kV. The nebulizer gas, collision gas, curtain gas, declustering potential, focusing potential, entrance potential, collision energy, and collision cell exit potential were set at the following values: 8, 4, 9, 41, 200–10, 30, 15, and 0, respectively^59^.

### UPLC-Q-TOF/MS analysis of blood samples after 4-EOTC treatment

The metabolomics analysis and data analysis were performed using UPLC-Q-TOF/MS. UPLC was performed in a Waters Acquity UPLC system (Waters, Milford, MA, USA) equipped with an autosampler and a binary solvent delivery system. The MS analysis was performed using a Waters Q-TOF Premier system (Micromass MS Technologies, Manchester, UK) equipped with an electrospray ionization (ESI) source operating in positive-ion mode. The nebulization gas was set to 600 L/h at a temperature of 350 °C, the cone gas was set to 50 L/h, and the source temperature was set to 100 °C. The capillary and cone voltages were set to 3 kV and 30 V, respectively. The Q-TOF acquisition rate was set to 0.2 s with a 0.01-s inter-scan delay. Argon was employed as the collision gas at a pressure of 7.066 × 10^−3^ Pa. Before sample testing, the TOF mass spectrometer was tuned and calibrated following the manufacturer’s instructions.

The UPLC-Q-TOF/MS data for all examined blood samples on day 16 were processed and analyzed using MarkerLynx (Waters, Milford, MA, USA) in the Masslynx software (version 4.1) for peak detection and alignment. For data collection, the parameters were set as follows: a retention time ranging from 1 to 10 min, a mass ranging from 100 to 2,000 Da, and a mass tolerance of 0.02 Da. Multivariate statistical analysis was performed using the EZinfo software, and the data were exported and analyzed using partial least squares discriminant analysis (PLS-DA). Pareto scaling was used in all the models to avoid chemical noise. Following these analyses, low-molecular-weight metabolites were detected as chromatographic peaks in the base peak intensity (BPI) chromatograms. Potential biomarkers were then selected according to their VIP values (VIP > 1.0), the loading plot and the S-plot. Some of these candidates could be identified by authentic standards, and the other potential biomarkers were interpreted according to available biochemical databases such as HMDB, Massbank and METLIN. Finally, the Kyoto Encyclopedia of Genes and Genomes, as well as the primary literature, were used to interpret the pathways associated with the biomarkers.

### Statistical analysis

All data are expressed as means ± s.e.m. unless otherwise indicated. Differences between two groups were assessed using the unpaired two-tailed Student’s t-test. Datasets that involved more than two groups were assessed by ANOVA, followed by Tukey’s post hoc test. In the figures, data with special markers are significantly different at *p* < 0.05, according to ANOVA statistical analyses performed by SPSS 22 software (SPSS Inc., USA). The results were considered statistically significant at *p* < 0.05.

## Additional Information

**How to cite this article**: Han, H. *et al.* Impact of 4-epi-oxytetracycline on the gut microbiota and blood metabolomics of Wistar rats. *Sci. Rep.* **6**, 23141; doi: 10.1038/srep23141 (2016).

## Supplementary Material

Supplementary Information

## Figures and Tables

**Table 1:** Potential biomarkers based on the UPLC-Q-TOF/MS analysis of blood samples from the treated groups compared with those from the control group after a 15-day 4-EOTC treatment.

Retention time (min)	*m*/*z*	Calculated mass (Da)	Elemental composition	Postulated identity	VIP values	Trend (L *vs*. C)	Trend (M *vs*. C)	Trend (H *vs*. C)
9.07	149.0315	148.0236	C_5_H_8_O_3_S	4-Methylthio-2-oxobutanoic acid	1.81	—	↓	↓
8.28	262.2497	261.2418	C_9_H_19_N_5_O_4_	Arginyl-serine	1.9	↓	↓	↓
8.21	218.2206	217.2127	C_10_H_23_N_3_O_2_	Deoxyhypusine	2	↓	↓	↓
4.72	205.0641	204.0562	C_7_H_12_N_2_O_5_	5-L-Glutamylglycine	1.46	↓	↓	↓
8.85	496.3472	495.3393	C_24_H_50_NO_7_P	LysoPC(16:0)	1.6	—	—	↓
8.83	518.3391	517.3312	C_26_H_48_NO_7_P	LysoPC(18:3)	1.32	—	↑	↑
10.07	546.3571	545.3492	C_28_H_52_NO_7_P	LysoPC(20:3)	2.24	—	↑	↑
8.58	544.3508	543.3429	C_28_H_50_NO_7_P	LysoPC(20:4)	1.86	↑	—	↑
8.86	587.3731	586.3652	C_37_H_62_O_5_	DG(34:5)	1.09	—	↑	↑
9.06	677.3856	676.3777	C_37_H_77_N_2_O_6_P	SM(d18:0/14:0)	1.77	—	↓	↓
9.05	721.4121	720.4042	C_38_H_73_O_10_P	PG(16:0/16:1(9Z))	1.92	—	↓	↓
9.98	781.4495	780.4416	C_45_H_85_N_2_O_6_P	SM(d18:0/22:3(10Z, 13Z, 16Z))	1.83	—	↓	↓
9.03	765.4434	764.4355	C_48_H_92_O_6_	TG(14:0/15:0/16:0)	2.01	—	↓	↓
7.70	793.459	792.4511	C_44_H_73_O_10_P	PG(16:1/22:6)	1.72	—	↓	↓

(Selected ion [M + H] +). C = control group, L = low-dose group, M = medium-dose group, H = high-dose group. ↑indicates significantly increased at *p* < 0.05; ↓indicates significantly decreased at *p* < 0.05 (one-way ANOVA); —indicates no statistical significance.

LysoPC: Lysophosphatidylcholine; DG: Diacylglycerol; SM: Sphingomyelin; TG: Triacylglycerol; PG: Phosphatidylglycerol.
