# Supplementary material for: Impact of 4-epi-oxytetracycline on the gut microbiota and blood metabolomics of Wistar rats
Source: Sci Rep. 2016 Mar 15;6:23141. doi: 10.1038/srep23141 (PMC4791543; doi:10.1038/srep23141)
Supplement: Supplementary Information [file srep23141-s1.doc]

**Impact of 4-epi-oxytetracycline on the gut microbiota and blood metabolomics of Wistar rats**

Hongxing Han^a^, Hailong Xiao^b^, Kai Zhang^a^, Zhenmei Lu^a*^

^a^ College of Life Sciences, Zhejiang University, 866 Yuhangtang Road, Hangzhou 310058, China; ^b^ Hangzhou Institute for Food and Drug Control, Hangzhou 310004, China

**Summary of supporting information**

1. **SUPPORTING FIGURES S1-S2**
   1. **Fig. S1** Rarefaction curves (a-d) and Shannon diversity index curves (e-h) of all sequencing samples in both genders.
   2. **Fig. S2** Cluster analysis and heat-map of all male (a) and female (b) fecal samples.
2. **SUPPORTING TABLES S1-S2**
   1. **Table S1** Primers of *tetQ*, *tetO* and 16S rRNA used in Q-PCR
   2. **Table S2** Phylogeny of the 43 key identified families (97% similarity level) between control and high dose groups on day 16.


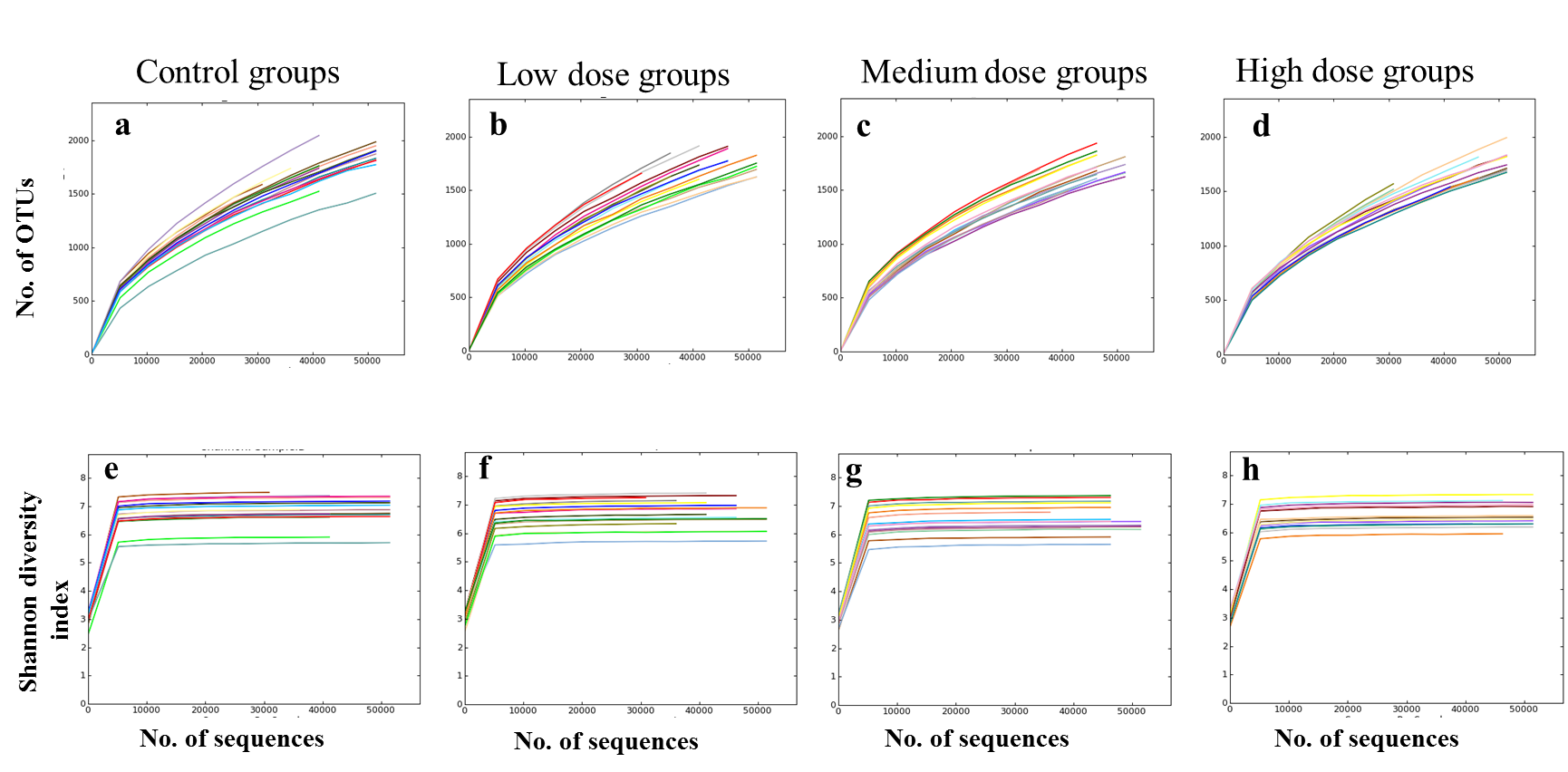


**Figure S1** Rarefaction curves (**a-d**) and Shannon diversity index curves (**e-h**) of all sequencing samples in both genders.


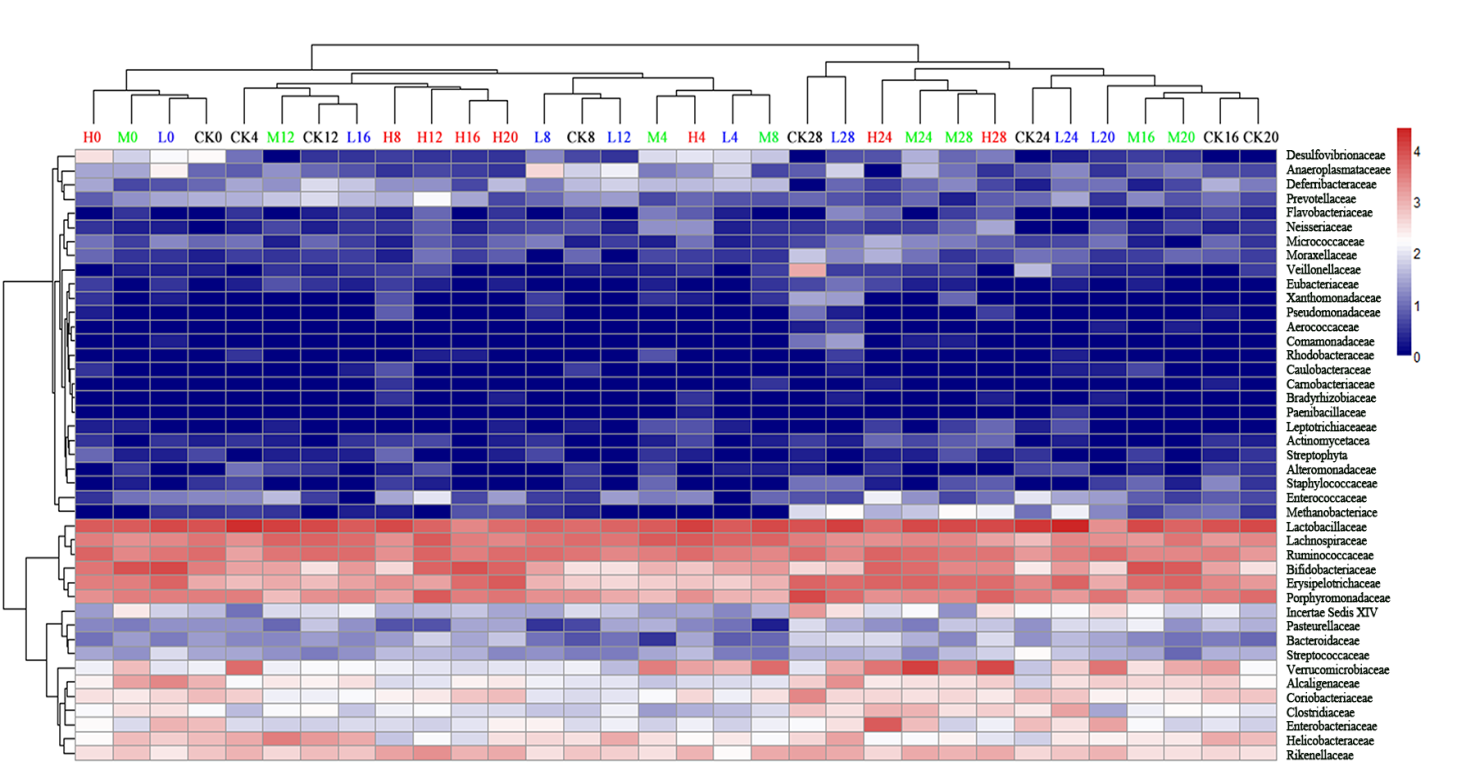


(**a**)


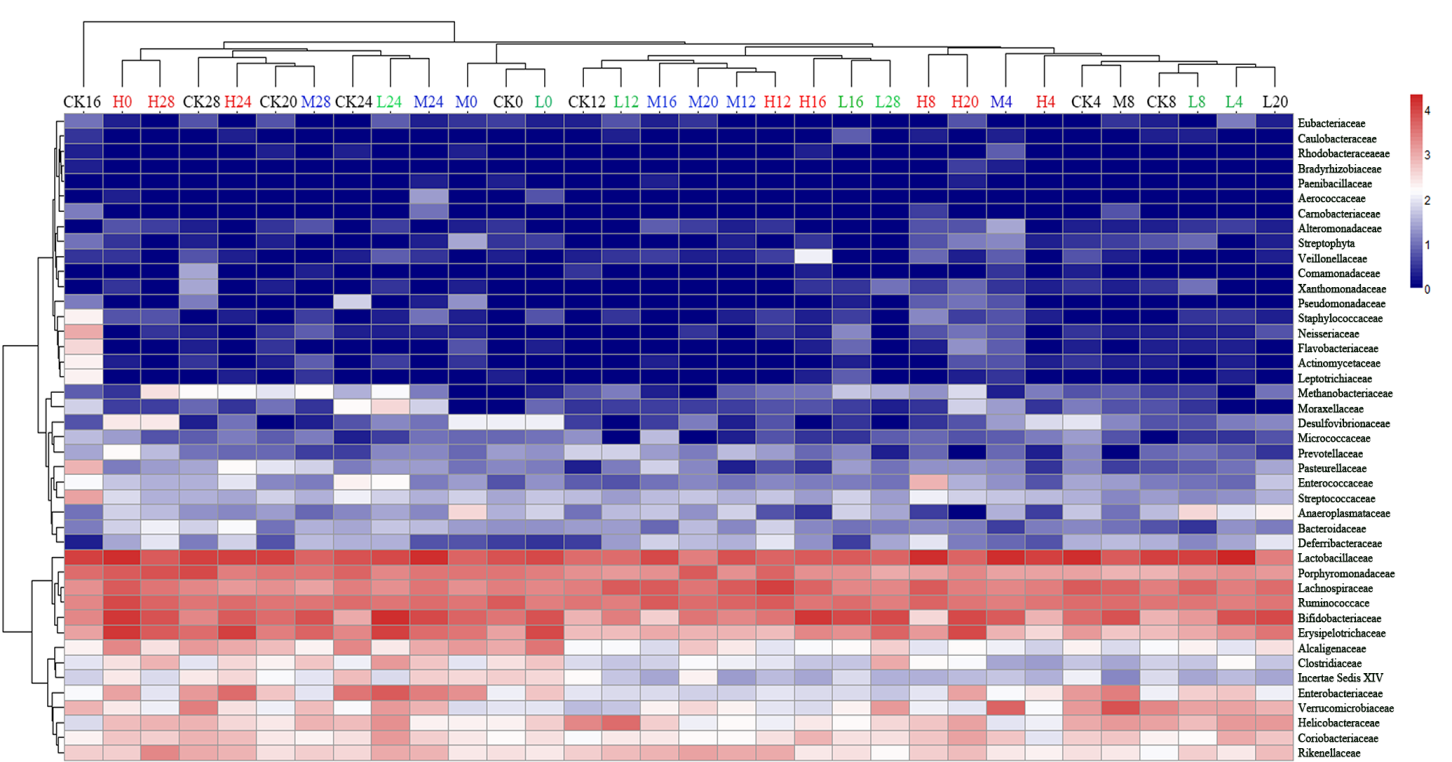


(**b**)

**Figure S2** Cluster analysis and heat-map of all male (a) and female (b) fecal samples. The font colors black, green, blue and red are control group (CK), low dose group (L), medium dose group (M) and high dose group (H), respectively. The number following the letter means the sampling time.

**Table S 1** Primers of *tetQ*, *tetO* and 16S rRNA used in Q-PCR

| Target gene | Primer sequence(5’-3’) | Product  size (bp) | | Annealing temperature (°C) | |  |
| --- | --- | --- | --- | --- | --- | --- |
| *tetQ* | F:GCTCACATTGATGCAGGAA | | 153 | 58 |  | |
|  | R:CGTAGAAGCCCGGACAGTAA | |  |  | | |
| *tetO* | F:GTGCCATCCTTGAGGAAAA | | 189 | 58 | |  |
|  | R:TGCTTTCATACTGCACTCCG | |  |  | | |
| 16S rRNA | F:CAGGAAACAGCTATGAC | | 131 | 55 | |  |
|  | R:GTTTTCCCAGTCACGAC | |  |  | | |

**Table S2** Phylogeny of the 43 key identified families (97% similarity level) between control and high dose groups on day 16.

**References**

1. O'Sullivan, L. A., Webster, G., Fry, J. C., Parkes, R. J., & Weightman, A. J. (2008). Modified linker-PCR primers facilitate complete sequencing of DGGE DNA fragments. *J. Microbiol. Methods*, *75*(3), 579-581.

2. Szczepanowski, R., Linke, B., Krahn, I., Gartemann, K., Guetzkow, T., Eichler, W., Puehler, A., & Schlueter, A. (2009). Detection of 140 clinically relevant antibiotic-resistance genes in the plasmid metagenome of wastewater treatment plant bacteria showing reduced susceptibility to selected antibiotics. *Microbiology*, *155*, 2306-2319.
